# A century‐long record of plant evolution reconstructed from a coastal marsh seed bank

**DOI:** 10.1002/evl3.242

**Published:** 2021-06-13

**Authors:** Michael J. Blum, Colin J. Saunders, Jason S. McLachlan, Jennifer Summers, Christopher Craft, Jeffrey D. Herrick

**Affiliations:** ^1^ Department of Ecology & Evolutionary Biology University of Tennessee Knoxville Tennessee 37996; ^2^ Southeast Environmental Research Center Florida International University Miami Florida 33199; ^3^ Department of Biological Sciences University of Notre Dame South Bend Indiana 46556; ^4^ School of Public and Environmental Affairs Indiana University Bloomington Indiana 47405; ^5^ U.S Environmental Protection Agency Office of Research and Development Research Triangle Park North Carolina 27711

**Keywords:** Chesapeake Bay, climate change, resurrection ecology, salinity, Schoenoplectus americanus, Scirpus olneyi, sea level rise

## Abstract

Evidence is mounting that climate‐driven shifts in environmental conditions can elicit organismal evolution, yet there are sparingly few long‐term records that document the tempo and progression of responses, particularly for plants capable of transforming ecosystems. In this study, we “resurrected” cohorts of a foundational coastal marsh sedge (*Schoenoplectus americanus*) from a time‐stratified seed bank to reconstruct a century‐long record of heritable variation in response to salinity exposure. Common‐garden experiments revealed that *S. americanus* exhibits heritable variation in phenotypic traits and biomass‐based measures of salinity tolerance. We found that responses to salinity exposure differed among the revived cohorts, with plants from the early 20th century exhibiting greater salinity tolerance than those from the mid to late 20^th^ century. Fluctuations in salinity tolerance could reflect stochastic variation but a congruent record of genotypic variation points to the alternative possibility that the loss and gain in functionality are driven by selection, with comparisons to historical rainfall and paleosalinity records suggesting that selective pressures vary according to shifting estuarine conditions. Because salinity tolerance in *S. americanus* is tightly coupled to primary productivity and other vital ecosystem attributes, these findings indicate that organismal evolution merits further consideration as a factor shaping coastal marsh responses to climate change.

**Impact Summary**

It is becoming increasingly evident that climate change can impose pressures that elicit organismal evolution, yet there are sparingly few long‐term records that document the tempo and progression of responses, particularly for plants capable of transforming whole ecosystems. In this study, we reconstructed a century‐long record of heritable responses of a foundational coastal marsh sedge to salinity exposure by conducting common garden experiments with age cohorts “resurrected” from a time‐stratified seed bank. We found that responses differed among revived cohorts, with plants from the early 20th century exhibiting greater salinity tolerance than those from the mid to late 20th century. The inferred rise and fall of salinity tolerance over time is analogous to evolutionary responses that have been observed in species like Darwin's finches that have been the subject of long‐term studies. It is also akin to records reconstructed for zooplankton and phytoplankton revived from lake and marine sediments, illustrating that soil‐stored seed banks can likewise serve as environmental archives that can yield new insight into the nature of plant evolution over century‐long time horizons.

Our findings also highlight the possibility that plant evolution may be a largely unrecognized or misattributed factor explaining variability in the structure and function of coastal marshes. Incorporating organismal evolution into predictive models might thus improve capacity to forecast and mitigate the consequences of climate change.

## Introduction

Evidence is mounting that climate warming and its corollaries can elicit organismal evolution. For instance, climate‐driven changes in seasonality have given rise to concomitant shifts in the photoperiodicity of pitcher‐plant mosquitos (Bradshaw and Holzapfel 2001). Indeed, it appears that even brief climate fluctuations can give rise to evolutionary responses, such as shifts in heritable phenological traits like flowering time in annual plants (Franks et al. 2007). Recent studies also have shown that evolutionary responses to climate‐related pressures can help maintain or alter ecosystem functionality, including vital processes related to productivity (Lohbeck et al. 2012; Schaum et al. 2017). Yet the tempo and progression of climate‐driven organismal evolution remain unclear, in part because there are few long‐term records of response that offer insight about the gain and loss of function over time.

Reviving long‐dormant propagules from natural and curated archives is proving to be a promising method for reconstructing decadal to century‐long records of evolutionary responses to environmental change, including changes associated with climate warming (Hansen et al. 2012; Geerts et al. 2015). For example, ephippia (i.e., resting stage eggs) of freshwater zooplankton recovered from time‐stratified lake sediments have been “resurrected” to reconstruct long‐term records of response to acidification, eutrophication, heavy metal contamination, and warming (Weider et al. 1997; Brendonck and De Meester 2003; Pollard et al. 2003; Derry et al. 2010; De Meester et al. 2011; Yousey et al. 2018). Similarly, sedimentary archives of marine diatom and dinoflagellate cysts have been utilized to reconstruct records of genetic variation and evolutionary responses to salinity, hydrographic, and temperature variation (Härnström et al. 2011; Ribeiro et al. 2013; Hinners et al. 2017; Ellegaard et al. 2018). Seeds also have been revived from stored collections to assess rapid evolution of plants in response to climate‐related drought (Franks et al. 2007; Franks and Weis 2008; Franks and Weis 2009; Franks 2011), and recent work (Summers et al. 2018) indicates that persistent and stratified soil‐stored seed banks can similarly serve as resources for examining ecological and evolutionary processes that shape plant populations over time.

In this study, we exploited a persistent soil‐stored seed bank of a Chesapeake Bay marsh to reconstruct a century‐long record of heritable variation in responses of a foundational sedge (*Schoenoplectus americanus*) to contrasting salinity conditions. We first assembled a century‐long sequence of “depth” cohorts, taking advantage of prior work on sediment stratigraphy and seed germination (Summers et al. 2018). We then conducted two common garden experiments to assay variation in growth and exposure responses to low and high salinity levels. Finally, to gain perspective on possible underlying mechanisms, we leveraged published accounts of relative abundance and multilocus genetic variation (Summers et al. 2018), paleo‐reconstructions (Cronin et al. 2000), and open access environmental records to explore how variation in salinity tolerance corresponds to shifts in genotypic composition, precipitation, and estuarine salinity conditions since the turn of the 20^th^ century.

## Materials and Methods

### STUDY SPECIES AND STUDY SITE

*Schoenoplectus americanus* is a foundational C_3_ perennial sedge that often dominates Gulf and Atlantic coastal marshes where mean salinity ranges between 3.5 ppt and 10.0 ppt (Smith 1995). Formerly known as *Scirpus olneyi*, *S. americanus* has emerged as a *de facto* model organism in studies of marsh responses to environmental change in part because vital ecosystem attributes like marsh surface elevation are tightly linked to trait variation and productivity (Ross and Chabreck 1972; Chabreck and Narcisse 1981; Rasse et al. 2005; Mueller et al. 2016; Lu et al. 2019). *S. americanus* is also of interest because it produces a prolific annual crop of seeds with exceptionally durable coats (Miller et al. 1997; Sherfy and Kirkpatrick 1999), which can result in highly stratified seed banks that persist for centuries (Brush 2001; Jarrell et al. 2016; Saunders 2003; Törnqvist et al. 2004). Accordingly, *S. americanus* seed banks have been used to infer shifts in genetic variation (Summers et al. 2018), relative abundance (Saunders 2003; Jarrell et al. 2016), ecosystem attributes (e.g., productivity, Saunders 2003), and ecosystem responses to sea level rise over time (e.g., Törnqvist et al. 2004).

All plants used in this study originated from Kirkpatrick Marsh, which is the site of the Global Change Research Wetland (GCReW) operated by the Smithsonian Environmental Research Center. The marsh, which supports a mixed C_3_‐C_4_ community predominantly of *S. americanus*, *S. patens*, and *Distichlis spicata*, borders the Rhode River, a sub‐estuary in the Chesapeake Bay (38^o^ 51″ N, 76^o^ 32″ W) near Edgewater, Maryland. Elevation of the marsh is 40 cm to 60 cm above mean low water, with 20% of high tides flooding the site (Jordan et al. 1986). The marsh has a mean salinity of 10 ppt, with growing season salinity ranging from 3 ppt to 15 ppt (Mozdzer and Caplan 2018). Interannual variation in growing season salinity is inversely correlated with rainfall (Saunders 2003), whereas multi‐decadal oscillations of estuarine salinity in Chesapeake Bay have corresponded to wet‐dry climate cycles over the last 500 years (Cronin et al. 2000). Like other areas in the Chesapeake Bay, Kirkpatrick Marsh is experiencing sea level rise at twice the rate as marshes in other embayments on the Atlantic Coast of North America (DeJong et al. 2015).

### SOIL EXCAVATION, RADIONUCLIDE ANALYSIS, AND SEED GERMINATION

In October 2002, we excavated a 30 cm diameter × 35 cm deep core (i.e., a “soil monolith”) to recover *S. americanus* seeds for germination assays, and in February 2004, we removed duplicate piston cores (15.2 cm diameter, 30 cm apart) to recover more seeds for germination assays and for radionuclide dating of soil strata. As described in Summers et al. (2018), seeds were sieved from the 2002 monolith and 2004 cores in 2 cm increments. Soil from one of the 2004 cores was dated by subjecting it to ^210^Pb and ^137^Cs radionuclide analysis, which allowed us to confidently constrain the age of seeds recovered from soil depths of ≤30 cm. Soil dates from ^210^Pb radionuclide data were estimated according to the constant rate of supply model (Appleby and Oldfield 1978), with variability in soil dates calculated by first‐order error analysis of counting uncertainty (Binford 1990). Peak ^137^Cs activity was also used as an independent marker of the depth corresponding to 1964, when ^137^Cs reached maximum concentrations in the atmosphere. We successfully germinated seeds recovered from 2 cm to 24 cm soil depths (Summers et al. 2018). Germination rates were statistically equivalent for soil depths above 14−16 cm, after which rates dropped by as much as 90% (Summers et al. 2018). The germination assays resulted in a total of 75 seedlings derived from 2 to 4 (2002 ±0.1), 8 to 10 (1984 ±1.2), 12 to 14 (1963 ± 3.0), 14 to 16 (1947 ± 4.2), 20 to 22 (1908 ±25), and 22 to 24 cm (1900 ±32.8) soil depths, which were propagated and maintained as stock plants for use in common garden experiments and genetic analyses (Summers et al. 2018). Hereafter we refer to each cohort according to the mid‐point calendar year derived from the estimated age of the respective soil layer.

### COMMON GARDEN EXPERIMENTS

We first conducted a limited scope, nonfactorial common garden study of plant growth following a germination trial (Summers et al. 2018). Using a total of 12 seedlings from the 1900, 1908, 1947, 1984, and 2002 cohorts, we measured the maximum stem height per pot (MaxHt) and change in MaxHt (*dMaxht* = ln (MaxHt_time2_) – ln (MaxHt_time1_)) at days 12, 19, 37, and 57 after planting. Light conditions were maintained at a 15:9 hr light to dark ratio, with temperature held at 30°C. From days 0–19 and days 38–57, the water level was maintained at <1 cm below the soil surface, whereas it was kept at >2 cm below the soil surface from days 19–37. Individual seedlings were not separated or transplanted to minimize mortality. We accordingly addressed the possibility of confounding effects from initial density and day of germination through statistical analyses described below.

We then conducted a two‐factor common garden study of growth responses to contrasting salinity conditions. Following Bennington et al. (1991) and Vavrek et al. (1991), we used tillers from stock plants for the experiment. Tillers included intact rhizome, stem, and root material, which was weighed wet and then planted in a 1:2 mixture of sand and peat soil. Six tillers (i.e., one per stock plant) were used for each of the 1947, 1984, and 2002 cohorts for each salinity treatment. Only five tillers were used for the 1908 cohort per treatment and only one tiller was used for the 1900 cohort per treatment due to limited availability of stock plants (Summers et al. 2018). Tillers were planted in separate pots arrayed in eight plastic tubs; four tubs were maintained at low salinity (LS, 3 ppt) and four were maintained at high salinity (HS, 15 ppt). Every week, the tubs were filled to just above the soil horizon with a mixture of water and Instant Ocean® sea salt (Aquarium Systems, Inc., Mentor, OH, USA) to maintain targeted salinity levels. Stem counts and heights were censused every week. After nine weeks, all plants were harvested and separated into live and dead aboveground (AG) biomass, as well as belowground (BG) rhizome and root biomass. All biomass compartments were weighed after being dried at 60°C.

### ANALYSIS OF GROWTH AND RESPONSES TO SALINITY CONDITIONS

For the non‐factorial experiment, cohorts were consolidated into genetically distinct “ancestral” (1900, 1908) and “descendant” (1947, 1984, 2002) groups to achieve sufficient replication (n = 7 and n = 5, respectively) (Summers et al. 2018). Repeated measures analysis of covariance (RM‐ANCOVA) was used to test for the main effects of group and day since planting, as well as group×day and group×water level interactions for MaxHt and *dMaxht*. Water level, initial seedling density, and day of germination were included as covariates.

For the two‐factor salinity experiment, the 1900 cohort was excluded from analyses because of insufficient replication. For the remaining four cohorts (1908, 1947, 1984, 2002), RM‐ANCOVA was used to analyze stem density and total stem length per pot for cohort, day, and salinity treatment main effects as well as cohort×day, cohort×salinity, and cohort×salinity×day interactions. Initial fresh mass was included as a covariate. When a main effect or interaction term was significant, a *post hoc* ANOVA was used to test for a significant cohort×salinity interaction by census date. Harvested biomass was analyzed with an ANCOVA to test the main effects of cohort and salinity, and for a cohort×salinity interaction. Dependent variables included total biomass, AG biomass, BG biomass, as well as rhizome biomass and root biomass. Initial fresh mass was included as a covariate. Where a main effect or interaction term involving cohort was significant, *post hoc* Least Squares means (LSM) tests were conducted to compare cohorts. The level of significance (i.e., the probability of Type I error, α) for LSM testing was Bonferroni corrected (α = 0.05/[*n*‐1], *p* < 0.0167) to account for multiple comparisons. All statistics were performed using SAS 9.1 (SAS Institute Inc., Cary, NC, USA).

### DEMOGRAPHIC, GENETIC, AND ENVIRONMENTAL CONTEXT OF SALINITY TOLERANCE

To gain perspective about the nature of temporal variation in exposure responses reconstructed from experimental findings, we used forward stepwise regression (Vellend et al. 2004, Blum et al. 2012) to examine how salinity tolerance corresponds to proxy measures of genotypic variation as well as historical precipitation and estuarine salinity conditions. For each cohort, we calculated the average and variance in treatment differences in total biomass (HS total biomass‐LS total biomass) to serve as measures of salinity tolerance. Taking advantage of prior work (Summers et al. 2018) demonstrating that persistent and stratified soil‐stored seed banks can serve as a resource for investigating genetic and demographic variation over time, we leveraged information on genotypic variation derived from Bayesian analysis of microsatellite loci scored for the plants used in the common garden experiments and plants from the 1963 cohort. For each cohort, we calculated the average and variance in proportional assignment to the two groups predominantly exhibited by the 1900 and 1908 ancestral cohorts (Summers et al. 2018) to serve as measures of genotypic variation. Average and variance estimates were calculated for measures of precipitation and paleosalinity for the time period corresponding to the 2 cm thickness of the sampled depth increment of each depth cohort. Measures of precipitation were derived from a composite historical rainfall record for the Baltimore‐Annapolis area (NOAA National Climatic Data Center) describing annual precipitation from the mid‐19th century to the late 20th century. Measures of paleosalinity were derived from estimates reconstructed for the mesohaline region of the Chesapeake Bay based on the stratigraphy of microfossils and pollen in radiometrically‐dated sediments (Cronin et al. 2000). Accordingly, we determined whether average genotypic composition, average precipitation, and average salinity were predictors of average salinity tolerance. We separately examined whether corresponding measures of variance were predictors of variation in salinity tolerance, and we assessed whether pairwise differences in genotypic composition, average rainfall, and average salinity were predictors of pairwise differences in average salinity tolerance among cohorts. A significance level of *p* = 0.10 was set for inclusion and exclusion of terms in regression models (Vellend et al. 2004, Blum et al. 2012). For further context, we leveraged a century‐long seed density profile (Summers et al. 2018) to draw comparisons to a proxy measure of relative abundance of *S. americanus* at the study site.

## Results

### GROWTH EXPERIMENT

We found that MaxHt varied over a three‐fold range at day 12, and over a twofold range thereafter (Figure S1). At all sampling dates, plants from the 1984 cohort exhibited the greatest MaxHt. After day 12, plants from the 1947 cohort exhibited the lowest MaxHt. We did not detect a significant main or interactive effect of group on MaxHt when cohorts were consolidated and analyzed as ancestral and descendant groups. However, we did detect significant group×day (*F*_1,19_ = 6.09, *p* = 0.0233) and group×water level interactions (*F*_1,19_ = 5.05, *p* = 0.0367) for *dMaxHt*, which was greater in the ancestral group from 19–37 days after planting, when water levels were lower (*post hoc* LS means comparison: *F*_1,20_ = 5.24, *p* = 0.0330; Figure S2). *dMaxHt* did not otherwise differ between the two groups. Only day of germination had a significant effect on *dMaxHt* (F_1,19_ = 12.85, *p* = 0.0059). Notably, the group×day and group×water level interactions were significant regardless of whether initial seedling density was included as a covariate in the model.

### SALINITY EXPOSURE EXPERIMENT

**Figure 1:**
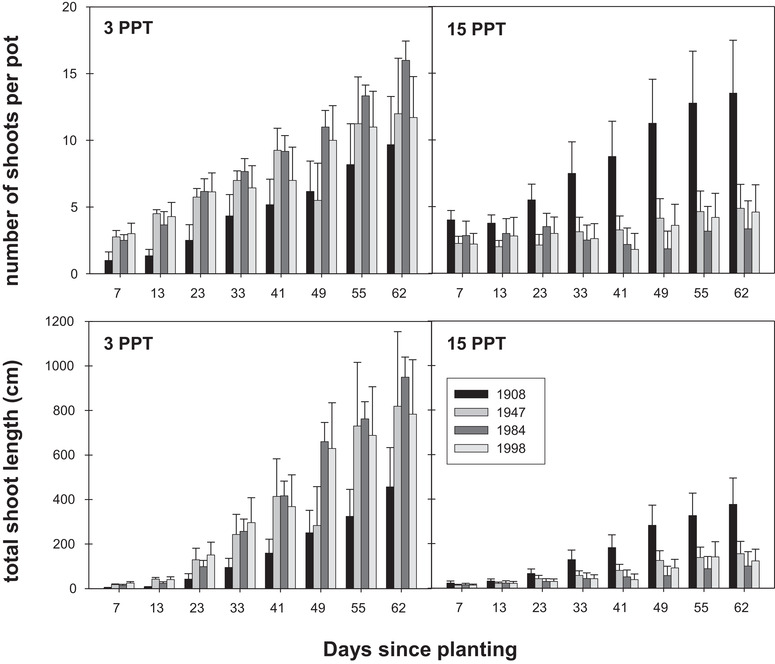
Shoot density (top panels) and total shoot length per pot (bottom panels) of *S. americanus* depth cohorts grown under low (3 ppt) and high (15 ppt) salinity conditions.

We found a significant cohort×day×salinity interaction for both shoot density (RM‐ANCOVA: *F*_24,304_ = 2.08, *p* = 0.0027) and total shoot length (*F*_24,304_ = 3.03, *p* < 0.0001), indicating growth responses to salinity differed among cohorts and that responses changed over the course of the experiment (Figure 1). *A post hoc* ANOVA of shoot density revealed significant cohort×salinity interactions (*p* < 0.05) for days 41–62. Although *post hoc* means comparisons failed to show significant differences among cohorts within a given treatment, the significant cohort×salinity interactions for days 41–62 reflected higher shoot densities of the 1908 cohort compared to the 1947 (+140 to +177%), 1984 (+57 to +513%), and 1998 (+83 to +386%) cohorts in the HS treatment (Figure 1) and lower shoot density of the 1908 cohort in LS treatment compared to the 1947 (+12 to −57%), 1984 (−39 to −60%), and 1998 (−18 to −59%) cohorts (Figure 1). Similarly, a *post hoc* ANOVA revealed significant cohort×salinity interactions for total shoot length (*p* < 0.05) for days 41–62. Total shoot length of the 1908 cohort was greater in the HS treatment (+41 to +398%) and lower in the LS treatment (−12% to −88%; Figure 1) relative to other cohorts. Initial mass did not have a significant effect on shoot density or length.

Though we did not detect a significant cohort×salinity interaction for measures of final biomass, differences among cohorts mirrored those found in the growth data (Table 1, Figure S2). The 1908 cohort consistently exhibited greater biomass in the HS treatment (+126 to +199%) and lower biomass (−67 to −75%) than all other cohorts in the LS treatment (Table 1). The largest among‐cohort differences were observed for rhizome biomass; the 1908 cohort had 462–847% more rhizome biomass than the 1947, 1984 and 1998 cohorts in the HS treatment, and 77–85% less biomass in the LS treatment (Table 1). Only root biomass in the 1908 cohort was not consistently greater (−22% to +54%) than the other cohorts in the HS treatment, although it was consistently lower (−75% to −76%) in the LS treatment (Table 1).

**Table 1:** Biomass (gdwt/pot) of *Schoenoplectus americanus* depth cohorts revived from seeds recovered from a Chesapeake Bay marsh seed bank and grown under low salinity and high salinity conditions, alongside a measure of salinity tolerance that reflects differences in response between HS and LS treatments. Values are means ± SE

Cohort (year)	Low salinity treatment	High salinity treatment	Salinity tolerance
*Total Biomass*			
1998	12.227 ± 4.126	0.839 ± 0.357	−11.388 ± 3.300
1984	13.031 ± 1.783	0.802 ± 0.526	−12.229 ± 1.373
1947	15.751 ± 5.849	1.060 ± 0.405	−14.691 ± 4.674
1908	4.008 ± 1.477	2.395 ± 0.810	−1.613 ± 1.299
1900	8.585 ± .	1.006 ± .	−7.579 ± .
*Live Shoot Biomass*			
1998	7.329 ± 2.389	0.572 ± 0.248	−6.757 ± 1.913
1984	8.582 ± 1.111	0.500 ± 0.319	−8.082 ± 0.854
1947	9.810 ± 3.519	0.773 ± 0.293	−9.037 ± 2.137
1908	2.958 ± 1.055	1.353 ± 0.421	−1.605 ± 0.906
1900	5.922 ± .	0.870 ± .	−5.052 ± .
*Dead Shoot Biomass*			
1998	0.246 ± 0.135	0.026 ± 0.019	−0.22 ± 0.108
1984	0.238 ± 0.080	0.040 ± 0.035	−0.198 ± 0.065
1947	0.340 ± 0.150	0.051 ± 0.030	−0.289 ± 0.094
1908	0.057 ± 0.032	0.080 ± 0.039	0.023 ± 0.037
1900	0.110 ± .	0.040 ± .	−0.07 ± .
*Rhizome Biomass*			
1998	2.647 ± 1.015	0.130 ± 0.104	−2.517 ± 0.813
1984	2.171 ± 0.518	0.087 ± 0.068	−2.084 ± 0.386
1947	3.628 ± 1.586	0.147 ± 0.076	−3.481 ± 0.959
1908	0.509 ± 0.263	0.826 ± 0.434	0.317 ± 0.360
1900	1.359 ± .	0.000 ± .	−1.359 ± .
*Root Biomass*			
1998	2.004 ± 0.698	0.111 ± 0.064	−1.893 ± 0.559
1984	2.041 ± 0.286	0.175 ± 0.141	−1.866 ± 0.247
1947	1.973 ± 0.724	0.089 ± 0.032	−1.884 ± 0.437
1908	0.484 ± 0.185	0.136 ± 0.059	−0.348 ± 0.156
1900	1.194 ± .	0.096 ± .	−1.098 ± .

### PREDICTORS OF SALINITY TOLERANCE

Forward stepwise regression revealed that average genotypic composition, average rainfall, and average estuarine salinity were not predictors of average salinity tolerance. Variation in rainfall (*t* = 8.98, *p* = 0.012) and variation in paleosalinity conditions (t = −3.365, p = 0.078) were retained as predictors in the regression model (full model: *R* ^2^ = 0.973, *p* = 0.023) of variation in salinity tolerance (Figure S3). Pairwise differences in genotypic composition (*t* = −13.952, *p* < 0.001), average rainfall (*t* = −10.935, *p* < 0.001), and average salinity (*t* = 15.855, *p* < 0.001) were retained as predictors in the regression model (full model: *R* ^2^ = 0.979, *p* < 0.001) of pairwise differences in average salinity tolerance among cohorts (Figure S3).

## Discussion

Our findings indicate that heritable variation in salinity tolerance in *S. americanus* has shifted in Kirkpatrick Marsh over the course of the 20th century. These results offer further evidence that evolution can occur on time scales congruent with unfolding trends in climate change (e.g., Rank and Dahlhoff 2002; Umina et al. 2005; Balanyá et al. 2006; Franks et al. 2007; Franks et al. 2014). It is also consistent with evidence that decadal‐long exposure to global change stressors elicits morphological adaptation in *S. americanus* (Lu et al. 2019). A congruent record of genotypic variation offers support for the inference that observed shifts in salinity tolerance reflect responses to selective pressures. Historical rainfall and paleosalinity records also suggest that shifting estuarine conditions could lead to the gain and loss of function due to variation in the strength of selection over time.

### A CENTURY‐LONG RECORD OF PLANT EVOLUTION

**Figure 2:**
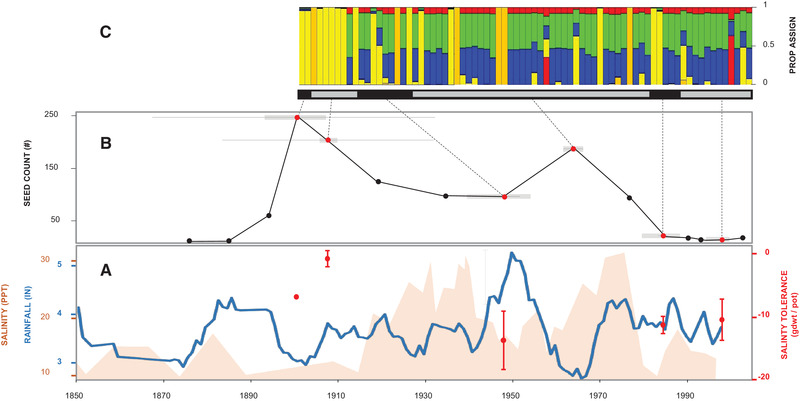
(A) *S. americanus* salinity tolerance (red filled circles and lines) versus a 9‐point smooth historic rainfall (IN = inches; blue line) compiled from Baltimore (1850‐1987) and Annapolis (1980‐1999) and paleosalinity (PPT = parts per thousand; pale orange fill) in Chesapeake Bay (*ca*. 1850–1996) redrawn from Cronin et al. (2000). (B) *S. americanus* seed abundance estimated from core 2004‐A plotted according to the depth interval midpoint ^210^Pb‐based age estimate (red circles) with reference to the age estimated for the top and bottom of the depth interval (grey bars) and the standard error of the midpoint age (horizontal black lines), with dates of soil depths >30 cm based on the mean accretion rate from 1868 to 1947 (0.23 cm/yr). (C) Bayesian proportional genotype assignments of *S. americanus* plants (K = 7) from six depth cohorts (differentiated according to alternating grey and black horizontal bars), including all cohorts used to estimate salinity tolerance (angled dashed black lines), redrawn from Summers et al. (2018).

Our results indicate that *S. americanus* exhibits heritable variation in responses to salinity exposure, and that salinity tolerance has shifted in the study population over the course of the 20th century. Our first experiment demonstrated that the pooled ancestral (1900‐1908) group exhibited higher growth than the descendant (1947‐1998) group under drier, less inundated conditions, which is suggestive of greater tolerance to stress. Consistent with this, our second experiment demonstrated that plants originating from the early 20th century exhibit higher salinity tolerance than those from the mid to late 20^th^ century (Figure 2, Figure S2). The inferred shifts in salinity tolerance could be an outcome of gene flow leading to a change in the composition of the study population over time. Evidence of strong genetic differentiation among neighboring marshes (Blum et al. 2010, Summers et al. 2018), however, does not support this hypothesis. Rather, it suggests that shifts in salinity tolerance are due to stochastic drift or natural selection. Other evidence also point to the possibility that shifts in salinity tolerance are an outcome of natural selection. Salinity stress, for example, can act as a selective agent (e.g., by altering nutrient availability and mineral uptake (Mitsch and Gosselink 2000)) capable of eliciting adaptive differentiation (Koehn et al. 1980; Purcell et al. 2008). Consistent with this, there is evidence that *S. americanus* seed production― a proxy measure of fitness― is negatively related to estuarine salinity conditions (Saunders 2003; Törnqvist et al. 2004; Jarrell et al. 2016). Elevated salinity also dampens *S. americanus* productivity and prevalence in coastal marsh communities (Erickson et al. 2007; Drake 2014; Jarrell et al. 2016), which underscores the potential value of further work on the fitness consequences of salinity exposure to clarify whether temporal shifts in tolerance reflect adaptive gain and loss of function over time.

Evidence of corresponding multi‐locus genotypic variation over time (Figure 2; Summers et al. 2018) provides additional support for the hypothesis that shifts in salinity tolerance are a product of natural selection (Merilä and Crnokrak 2001). Pairwise differences in genotypic composition were, for example, recovered as a predictor of pairwise differences in average salinity tolerance among depth cohorts. While this statistical finding should be viewed with some caution due to low degrees of freedom (i.e., because inferential power is limited by small sample sizes (Button et al. 2013, Wasserstein and Lazar 2016)), it is notable that plants in the 1900 and 1908 cohorts exhibit higher salinity tolerance also exhibit distinct Bayesian genotype assignment profiles relative to the profiles of plants in more modern cohorts (Figure 2). It is also notable that the 1947 cohort, which is composed of plants that collectively exhibit the greatest range of salinity tolerance, also exhibits a transitional composition, encompassing a mixture of genotypes (Figure 2). Further work on the functional basis of genotype‐phenotype associations, perhaps focusing on measures of genome‐wide or transcriptional variation, might reveal stronger signatures of natural selection.

Historical records and paleo‐reconstructions convey that there has been considerable change in precipitation and estuarine conditions since the mid 19th century (Figure 2; Cronin et al. 2000), raising the possibility that selective pressures have varied over time.

Although it was not possible to undertake comparisons to environmental conditions at particular time points, consideration of broader trends revealed intriguing evidence of concordance between changes in salinity tolerance, precipitation, and salinity over time (Figure S3). Yet some notable departures are also evident, where levels of salinity tolerance do not align with trends in precipitation or paleosalinity (Figure 2). For example, cohorts from the mid to late 20th century exhibiting lower salinity tolerance coincide with periods of high and moderate rainfall respectively, but older cohorts exhibiting higher salinity tolerance appear to have originated after a period of lower rainfall (Figure 2). Instances of temporal discordance (i.e., lags) between trait and environmental variation (Figure 2) point to the possibility that plasticity moderates the tempo and synchronicity of evolutionary responses of *S. americanus* to episodes of stressor exposure inferred from environmental records (Jump and Peñuelas 2005; Gienapp et al. 2008). Discordance might also arise because population‐scale measures of salinity tolerance reflect non‐additive responses to other selective agents (e.g., inundation, rising atmospheric CO_2_) or constraints (Davis et al. 2005). Additionally, discordance might be an outcome of interactions among overlapping generations resulting from prolonged longevity or recruitment from the soil‐stored seed bank, which can act as a reservoir of (mal)adaptive variation (Hairston and De Stasio 1988; Hairston 1996; Summers et al. 2018). Future reconstructions of plant responses to environmental change should consider drawing comparisons based on local environmental records as well as addressing sources of uncertainty in analyses that rely on sediment chronologies and paleo‐reconstructions. Drawing comparisons across additional levels of stressor exposure or a larger number of depth cohorts that span longer time horizons circumscribing sharply contrasting conditions (e.g., Frisch et al. 2014) could also provide a stronger basis for testing hypotheses and interpreting reconstructed records of organismal responses to environmental change.

### ECOSYSTEM OUTCOMES OF ORGANISMAL EVOLUTION

Evidence is mounting that evolution can be an important determinant of how ecosystems function (Whitham et al. 2006, Schaum et al. 2017; Monroe et al. 2018; Ware et al. 2019). Our findings shed further light on how evolution can engender ecological change. Salinity tolerance is tightly coupled to *S. americanus* growth and primary productivity (Ross and Chabreck 1972; Saunders 2003; Rasse et al. 2005), thus it is possible that associated ecosystem attributes are subject to evolutionary responses to salinity stress. Evolution might govern marsh platform elevation, for example, because *S. americanus* responses to salinity involve shifts in functional traits, such as root structure, stem density, and canopy height, that determine soil organic matter accumulation and mineral deposition (Figure 1; Leonard and Luther 1995; Christiansen et al. 2000; Seliskar et al. 2002; Bernik et al. 2018; Lu et al. 2019). Because the sum of these effects can be large (Baustian et al. 2012), even marginal evolutionary changes in the capacity of *S. americanus* to accommodate salinity stress could have pronounced aggregate impacts on marsh ecosystems. Thus organismal evolution may very well be a largely unrecognized or misattributed factor explaining variability in the structure, function, and fate of coastal marshes (Lu et al. 2019).

## Conclusions

This study further illustrates how persistent and stratified soil‐stored seed banks can serve as valuable archives for studying responses of plants to environmental change (Summers et al. 2018). The inferred rise and fall of salinity tolerance over time in the study population of *S. americanus* is similar to decadal‐scale patterns of evolution observed in species like Darwin's finches (Grant and Grant 2002) that have been the subject of long‐term field studies. It is also akin to records reconstructed for zooplankton and phytoplankton revived from lake and marine sediments (Weider et al. 1997; Brendonck and De Meester 2003; Derry et al. 2010; Härnström et al. 2011; Ribeiro et al. 2013; Frisch et al. 2014; Geerts et al. 2015; Yousey et al. 2018), demonstrating that retrospective “resurrection” approaches can yield new insight into the nature of ecological and evolutionary processes that shape plant populations over time. By focusing on heritable trait variation in a foundational species, we also have illustrated that it can offer novel perspectives on ecosystem outcomes of environmental change. As soil‐stored seed banks are a largely untapped resource for scientific inquiry, further work to improve their use for retrospective “resurrection” studies could substantively advance understanding of linkages between the evolution of constituent species and ecosystem attributes of coastal marshes and other ecosystems of interest. Key next steps include reducing uncertainty in sediment age estimates and minimizing discontinuities by reconstituting larger cohorts from finer scale depth intervals. Developing pedigreed offspring from plants originating from revived seeds would eliminate possible residual influences of long‐term burial and maternal effects (Summers et al. 2018; Weis 2018) and enable the analysis of trait heritability and the association of traits with fitness (Franks et al. 2007). These and other potential advances (Summers et al. 2018) could offer a stronger basis for incorporating organismal evolution into predictive models to improve forecasts of ecosystem function (e.g., C cycling) and fate (e.g., drowning) under near‐future scenarios of global change.

## AUTHOR CONTRIBUTIONS

M.B., C.S., J.M., and J.H. conceived and designed the study, C.S. and C.C. collected the data, M.B., C.S., C.C., J.M., and J.S. analyzed the data, M.B., C.S., J.M., and J.H. drafted the initial version of the manuscript and all authors contributed to later versions of the manuscript.

## DATA ARCHIVING

All data supporting the results of this study have been deposited in Dryad (https://doi.org/10.5061/dryad.7m0cfxptt).

## Supporting information


**Figure S1**. Maximum stem height (MaxHt) (top graph) and change in MaxHt (bottom graph) of *S. americanus* depth cohorts grown in the non‐factorial common garden experiment.
**Figure S2**. Boxplots of total (aboveground + belowground) biomass of *S. americanus* depth cohorts under low and high salinity conditions. Thick lines and thin lines indicate the mean and median biomass, respectively.
**Figure S3**. Three‐axis scatterplots with a linear surface depicting statistically significant relationships between (A) variance in salinity tolerance, rainfall, and estuarine salinity; (B) pairwise differences in salinity tolerance, rainfall and estuarine salinity; and (C) pairwise differences in salinity tolerance, genotypic composition and estuarine salinity. Δ = pairwise difference.
