# Supplementary material for: A century‐long record of plant evolution reconstructed from a coastal marsh seed bank
Source: Evol Lett. 2021 Jun 13;5(4):422–31. doi: 10.1002/evl3.242 (PMC8327947; doi:10.1002/evl3.242)

*Evolution Letters*

Supporting Information for

**A century-long record of plant evolution**

**reconstructed from a coastal marsh seed bank**

**FIGURES**

**Figure S1.** Maximum stem height (MaxHt) (top graph) and change in MaxHt (bottom graph) of *S. americanus* depth cohorts grown in the non-factorial common garden experiment.


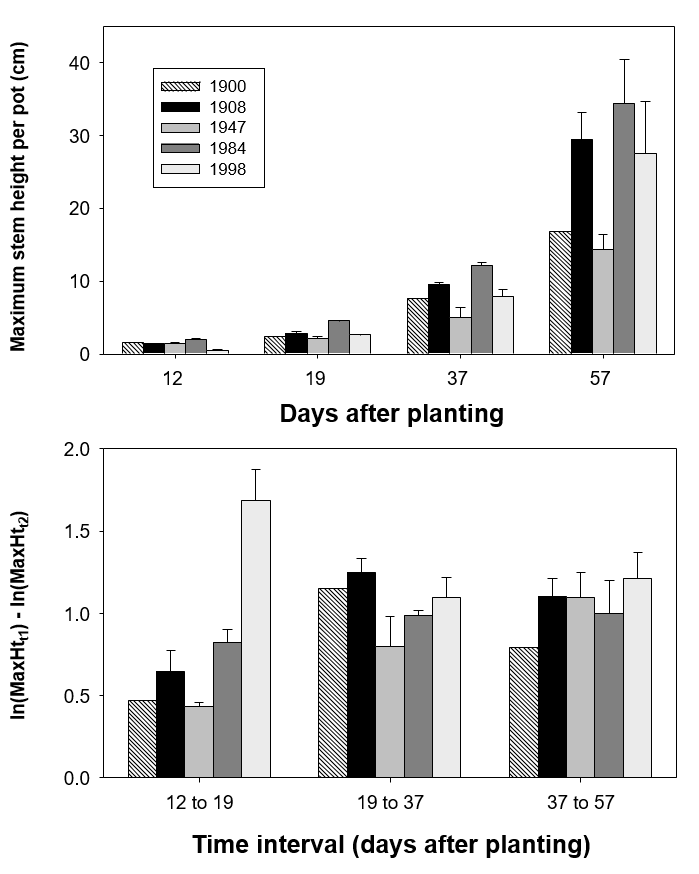


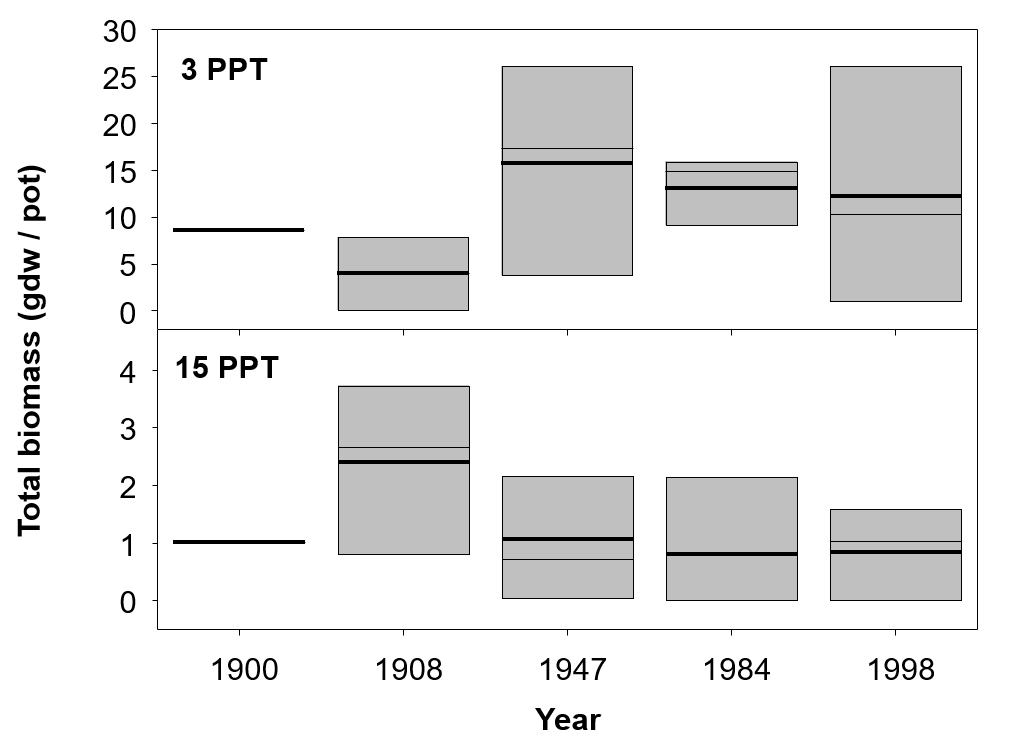
**Figure S2.** Boxplots of total (aboveground + belowground) biomass of *S. americanus* depth cohorts under low and high salinity conditions. Thick lines and thin lines indicate the mean and median biomass, respectively.

**Figure S3**. Three-axis scatterplots with a linear surface depicting statistically significant relationships between (A) variance in salinity tolerance, rainfall, and estuarine salinity; (B) pairwise differences in salinity tolerance, rainfall and estuarine salinity; and (C) pairwise differences in salinity tolerance, genotypic composition and estuarine salinity. Δ = pairwise difference.


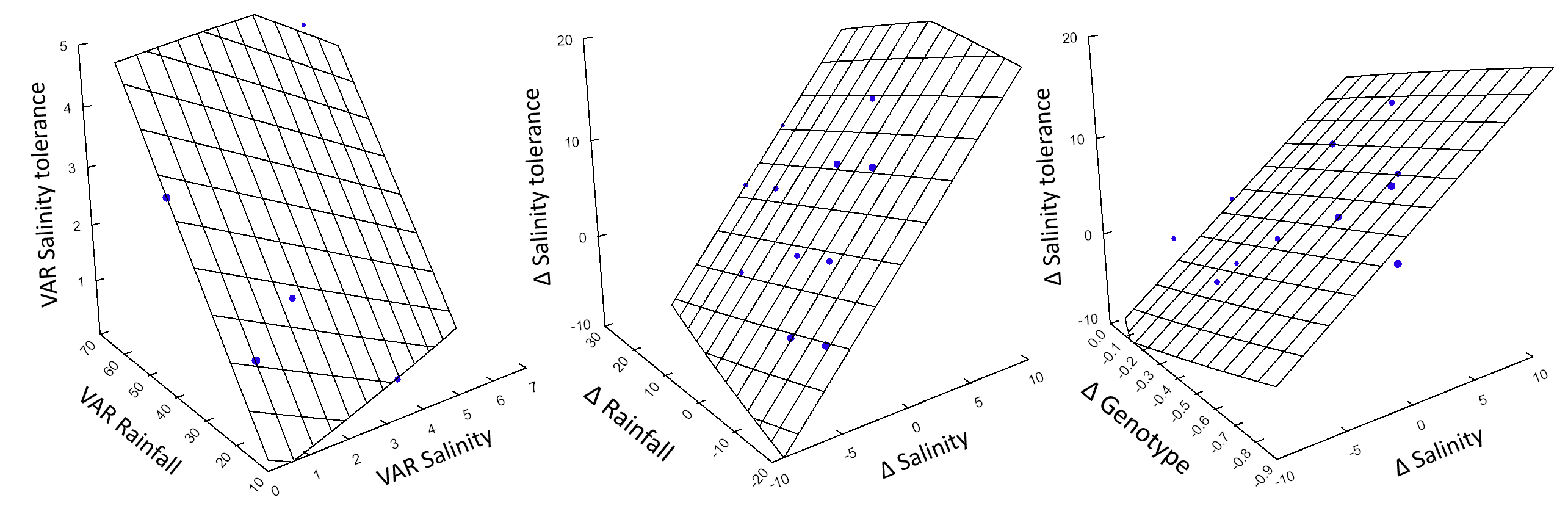

Supplement: Supplementary file 1 — Figure S1. Maximum stem height (MaxHt) (top graph) and change in MaxHt (bottom graph) of S. americanus depth cohorts grown in the non‐factorial common garden experiment. Figure S2. Boxplots of total (aboveground + belowground) biomass of S. americanus depth cohorts under low and high salinity conditions. Thick lines and thin lines indicate the mean and median biomass, respectively. Figure S3. Three‐axis scatterplots with a linear surface depicting statistically significant relationships between (A) variance in salinity tolerance, rainfall, and estuarine salinity; (B) pairwise differences in salinity tolerance, rainfall and estuarine salinity; and (C) pairwise differences in salinity tolerance, genotypic composition and estuarine salinity. Δ = pairwise difference. [file EVL3-5-422-s001.docx]
